# Does the Legal Framework of the United Arab Emirates Comply with International Standards for the Protection of Foreign Investors?

**DOI:** 10.12688/f1000research.184809.1

**Published:** 2026-07-04

**Authors:** Nagwa Abouhaiba, Said Saddiki, Ahmed Elsawi, Maya Khater

**Affiliations:** 1 Abu Dhabi University, Abu Dhabi, United Arab Emirates; 2 Sidi Mohamed Ben Abdellah University, Fez, Morocco; 3 Shura law Firm for Advocacy and Arbitration, Cairo, Egypt; 4 United Arab Emirates University, Al Ain, Abu Dhabi, United Arab Emirates

**Keywords:** Foreign Investor Protection, International Investment Law, International Investment Arbitration, Fair and Equitable Treatment, UAE Investment Legislation.

## Abstract

The protection of foreign investors has witnessed significant development under international investment law, particularly with the increase of bilateral investment agreements, and the different mechanisms of international investment arbitration. Despite the development of international standards for protecting foreign investors, there is still ongoing legal and arbitration debate about whether the national laws of host states are fully consistent with these standards or not. This study discusses the legal framework that is regulating the protection of the foreign investors in the United Arab Emirates, by analyzing the relevant UAE legislation, including key investment protection standards. In addition, this study discusses the practical challenges resulting from the interaction between the national legal system and international investment arbitration, particularly with regards to the different interpretations of investment protection standards, and the wide discretionary powers granted to arbitration tribunals. This study adopts a comparative analytical approach, supported by a critical analysis of selected arbitration awards. This was achieved through examining the rules regulating the protection of foreign investors under UAE legislation and comparing them with the relevant international standards, while also benefiting from comparative models, particularly those of France and Singapore. The study concludes that there is a relative degree of integration between the UAE’s legislations and the principles of international investment law. The study also shows that the UAE legal system has achieved noticeable progress in strengthening the investment environment. However, some aspects still require more legislative clarity and better regulatory consistency with contemporary standards of international investment law. Accordingly, this study recommends further development of legislative and regulatory mechanisms related to foreign investment, in order to enhance consistency with international investment standards.

## Introduction

The protection of foreign investors is considered one of the most important topics in modern law because it has a direct impact on attracting foreign investments, supporting economic activity, and achieving commercial stability ( Lashchykhina & Lashchykhina, 2024). Therefore, many countries, including the UAE, are working on developing their legislation in a way that matches the requirements of international investment and provides a level of legal stability for foreign investors. These countries are also expanding their participation in international investment protection agreements, whether bilateral or multilateral, which include legal standards such as fair and equitable treatment and the prohibition of expropriation without fair compensation ( UNCTAD, 2023).

This caused stronger interaction between the national legal rules, and the rules of the international investment law, especially with the growing role of the international investment arbitration as a major method for disputes resolution between the investors and the host states ( Marcoux et al., 2024).

The development of the UAE’s legislation has helped in strengthening the state’s ability to keep up with the modern legal challenges, which has improved its position as an important investment destination at both the regional and international levels ( Khater et al., 2025b; AlQodsi et al., 2026). In particular, the issuance of the UAE’s Civil Transactions Law No. 25 of 2025 has raised new questions about whether traditional legal rules are sufficient to keep pace with the rules of international investment law, especially the principles that are applied in the disputes resolution process before the International Center for Settlement of Investment Disputes ( ICSID, 1965).

This raises important questions about the capability of the national legal rules in providing effective protection for foreign investors according to the modern international standards.

The importance of this question has increased because of the economic and regional changes that the Gulf region has experienced in recent years. These Changes have led some international markets to become more cautious when evaluating investment opportunities and making decisions related to long term investments. As a result, the need for a clear and stable legal system has became more important in order to provide the foreign investors with the necessary protection and help in maintaining the legal and economic stability of the investments parties in a rapidly changing economic and regional environment.

Although the UAE Civil Transactions Law is based on general principles such as good faith, freedom of contract, and the obligation to perform contractual commitments, as it is considered the main law regulating the contractual relationships, including the contracts related to investments, this makes it a general legal framework that provides indirect protection for foreign investors ( Al Tamimi, 2020; Khater et al., 2025a; El-Erian et al., 2026). However, the effectiveness of this protection still needs practical evaluation when it is compared with the standards applied in international investment arbitration cases.

Based on the above, the purpose on this research is to evaluate how effective the legal protection provided by UAE legislation is for foreign investors through a comparative study with international protection standards, while also analysing some practical applications related to international investment arbitration. The research also aims to provide a comparative critical analysis that explains the similarities and differences between national legal rules, and international investment protections standards, and shows the extent to which UAE legislation is consistent with modern developments in international investment law.

Accordingly, the main issue of this study is to evaluate the extent to which the UAE legal framework of protecting foreign investors is consistent with modern standards in international investment law, and whether this framework is capable of achieving a balance between the protection of investors and the preservation of the state’s national sovereignty considerations.

In this context, the main research problem of the study is to assess how far the UAE’s legal system is aligned with the modern standards on international investment law in regard to protecting the foreign investors, and to examine its ability to balance investor protection with the state’s regulatory sovereignty. Based on the economic and regional changes that the Gulf region has experienced in recent years, this issue has become more important, along with the reconsideration of some of the investment decisions, and the increased level of scrutiny by the international investors regarding the stability of the legal and economic environments. These developments raised questions about whether the current legal protection is sufficient and effective in responding to such changes and maintaining the stability of the long-term investment relationships.

The importance of this study lies in the fact that it does not only describe or present legal rules, but also focuses on the analysis, the comparison, and the constructive criticism to examine whether the protection that is provided by the national legal system is sufficient when it is compared with the international standards, and to determine whether this protection achieves the required level of effectiveness in the practical application or not.

The importance of this study also appears in explaining the similarities and the differences between the UAE’s civil law, and the international investment law, which helps in improving the understanding of the complementary connection between the two systems, and recognizing the impact of this relationship on the position of the foreign investor. This is especially important because the international arbitration tribunals often rely on standards that may differ in the interpretation and the application from the national legal rules.

Therefore, this study contributes in providing an analytical perspective that helps in achieving a deeper understanding of the connection between the legislative sovereignty of the state, and the requirements of the international investment system, in a way that supports the developments of the investment legal environment in the UAE with a greater balance and clarity.

In addition, this research makes a contribution in enriching the legal scholarship and studies related to the protection of the foreign investment, by providing a systematic analysis that helps researchers and decision makers understand the nature of the legal protection and how it develops as a result of the interaction between the national law, and the international law.

The problem of protecting foreign investors has recently received significant attention in modern legal studies, especially with the expansion of the international investment activities and the increasing reliance on the investment arbitration, as a main method for disputes resolution between the foreign investors and the host states. Previous studies have discussed this topic from different perspectives, including the guarantees provided by international investment law, the role of national legislation in providing legal protection for investors, and the impact of international arbitration in defining the scope of this protection and applying in practice.

In addition, reports issued by the United Nations Conference on Trade and Development ( UNCTAD, 2023) have pointed out that investment arbitration has become an essential part of the practical application of international investment law. At the same time, a legal debate continues regarding the extent to which a balance can be achieved between protecting foreign investors on one side and the preservation of the state’s right to regulate its economic and legislative policies on the other side ( Van Harten, 2020; Sornarajah, 2021).

With regard to the UAE’s legislation, the study by Al Tamimi 2022 discussed the legal protection provided to foreign investors in the UAE, especially in the light of the Foreign Direct Investment law No. 19 of 2018. The study concluded that the UAE’s legislation includes several legal and procedural guarantees that provide an important level of protection for foreign investors, whether in terms of freedom to carry out economic activities or the protection of funds and investments.

However, the study also pointed out that there are some practical and regulatory issues that may affect the actual level of protection when compared with international standards, especially regarding the clarity of investment disputes settlement mechanisms, and their consistency with modern international trends. This point was also confirmed by the study of Marcoux et al. (2024).

In comparative studies, Dolzer & Schreuer (2012) discussed the relationship between national legislation and international investment treaties. The study concluded that the international agreements usually have clearer and more specific standards of protection, especially regarding the principle of fair and equitable treatment and the protection against arbitrary measures. It also explained that the differences between the application of the national rules, and the modern trends in investment arbitration may lead sometimes to different interpretation of investment protection standards. This may affect the legal clarity level and the stability of the investment relationships, which is in the same direction that was highlighted in the Peat et al. (2025) analysis.

Through reviewing these studies, it becomes clear that they importantly contributed to analyzing the different aspects of the foreign investors’ protection. However, most of them made the discussion on the national and international aspects separate from each other. Or it focused mainly on the investments arbitration as a method of dispute settlement, without directly evaluating the adequacy and effectiveness of the protection provided by the UAE’s legal system to foreign investors when compared with modern standards of international investment law, especially in the light of the practical application of investment disputes and the development of the international arbitration trends.

Therefore, this research seeks to fill this research gap through a comparative analytical study of the adequacy and effectiveness of foreign investors protection in the UAE. This will be done by evaluating the extent to which national legislation is consistent with modern standards of international investment law and by analyzing the level of legal protection provided in practical application, in order to present a clearer evaluation of how far the UAE’s legal system responds to the requirements of international investment.

## Methods

This study depends on the comparative analytical approach, as it is considered the most suitable method for the nature of this research topic, which focuses on evaluating the effectiveness of the foreign investors protection, and the UAE, and the examination of the extent to which the national legislation is consistent with the modern standards in the international investment law.

The analytical approach is based on the examination of the legal provisions that are regulating foreign investments under the UAE’s legislation, especially the civil transaction law No. 25 of 2025, the commercial companies law, and the UAE’s foreign direct investment law. It also includes analysis of the main principles of international investment law, particularly the principle of fair and equitable treatment, the protection against unlawful expropriation, and the mechanisms used for settling international investment disputes.

In addition, this study also adopts the competitive approach by benefiting from some comparative models, especially the French, and Singaporean experiences, with the aim of evaluating whether the protection provided by the UAE’s legal system is sufficient, as well as identifying its strengths, and the aspects that still require further development in light of modern trends in the international investment law.

This study also uses critical analysis when discussing some of the practical applications used in decision making by international investment arbitration tribunals, in order to evaluate the consistency of the arbitration interpretations with the national legal rules and to explain the impact of this on the actual level of the protection provided to the foreign investors.

## Results

This study concluded that the legal protection provided by the UAE’s national law for foreign investors achieves a suitable level of effectiveness in regulating the investment relationships with contractual nature. However, this protection becomes more effective when combined with the specialized guarantees provided by the international investment law. This study also showed that the relationship between the national and international systems is not based on conflict, but rather on a level of legal integration. Where the UAE legislation provides the legal foundation for the stability of investment transactions, while international investment law offers more specialized guarantees in the field of investment protection and dispute settlement.

This study shows that the UAE’s Civil Transactions Law No. 25 of 2025 includes several important legal principles, especially the principles of good faith, the freedom of contract, and the binding force of contracts. These principles strengthen confidence in civil and investment transactions and support the legal stability of contractual relationships related to investment activities. This study also found that the modern UAE’s legislation, especially the foreign direct investment law and the commercial companies law, has significantly contributed to improving the attractiveness of the investment environment by expanding foreign ownership opportunities, simplifying regulatory procedures and providing financial and procedural guarantees for foreign investors.

On the other hand, the study reveals that international investment law provides foreign investors with more specialized protection through a set of substantive standards regulating investment protection, such as the principle of fair and equitable treatment and protection against unlawful expropriation. In addition, the study allowed the use of international investment arbitration as an effective method of investment disputes resolution and strengthened confidence in international transactions.

Some challenges that are related to the interaction between the national and the international legal systems are highlighted in this study. One of the main challenges is the existence of multiple laws that are regulating foreign investment, along with the absence of unified legislative body of definitions, for some legal concepts related to investment protection. In addition, there is a relative difference in the interpretation of some international protection standards, within the international investment arbitration, Especially the principle of Fair and equitable treatment. This study also shows that the wide discretionary powers that are given through the tribunals of arbitration may sometimes lead two different outcomes in disputes that involve similar facts, which raise issues related to legal stability and the predictability of legal positions.

This study confirms that the main issue is not the absence of legal protection for foreign investors, but rather the adequacy and effectiveness of this protection in practical applications. This issue becomes more important in the light of the economic and regional changes that the gulf region has experienced in recent years, as well as the tendency of some investors and international institutions to reassess the level of risks associated with long term investments and the stability of the legal environments governing them.

In this context it is important to examine whether the current legal protection level Is capable of responding to this changing circumstances. This requires considering the interaction between the national legal system and the rules of the international investment law, as differences in interpretation or the practical difficulties and applying certain investment protection standards may arise. Therefore, there is I need for a greater legislative clarity regulatory consistency to ensure stability in the legal protection for foreign investors, and to strengthen the confidence in the investment environment in the UAE.

## Discussion

The issue of how far the UAE legal framework is consistent with international standards for protecting foreign investors has become increasingly important due to the interaction between national legal rules and the rules of international investment, especially with the growing number of international investment disputes and the increasing reliance on international investment arbitration.

A foreign investor refers to a natural or legal person belonging to another country who invests capital or economic resources in the host state with the aim of achieving an economic return. The foreign status of the natural person is determined according to the nationality criterion, while for companies it is usually determined according to the place of incorporation or the place of management ( Sachs, L., Johnson, L., & Coleman, J. 2022).

Some modern legislation, including UAE’s legislation, have adopted a more flexible concept of the foreign investors. This can be seen in the Foreign Direct Investment Law, where article 1 defines the foreign investor as: “A natural or corporate person who does not hold the nationality of the State and who invests in the State in accordance with the provisions of this Law by Decree” ( UAE Federal Law No. 19 of 2018).

Foreign investment refers to the transfer of capital or economic resources from one country to another for the purpose of achieving an economic return.

Legal scholars usually divide foreign investment into two main types:

• Foreign Direct Investment (FDI): This type of investment exists when the foreign investor owns an effective and influential share in a project within the host state and also participates in its management or supervision.
• Indirect Foreign Investment: This type of investment is limited to purchasing shares and bonds without actual participation in the management of the project ( UNCTAD, 2023).

From the above, it becomes clear that the concept of foreign investor is related to the status of the investing person, whether through the existence of a foreign element or foreign nationality regarding the ownership of capital. On the other hand, the term foreign investment refers to the process of investing capital in a country different from the nationality of the investor with the aim of achieving an economic return.

### Legal protection of foreign investors under UAE legislation now

With the issuance of the UAE civil transactions law No.25 of 2025, the UAE legislator moved toward regulating contractual relationships in a more flexible and stable manner, including contracts in which one of the parties is a foreign investor. This can be seen through the strengthening of the rules governing the performance of contractual obligations and compensation for breach of contract, which helps provide high level of legal stability and confidence in investment transactions. As a result, civil law has gained an important role in evaluating the effectiveness of the legal protection granted to foreign investors within the state, especially in investment contracts and the rights and obligations related to them.

The law also established the principle of good faith as a rule governing contractual relationships at all stages ( Chami et al., 2025). This principle is no longer limited only to the stage of contract performance, but now also includes the stages of negotiation, conclusion, and implementation of the contract. This reflects A broader approach in regulating contractual relationships, as it provides protection against arbitrary or unlawful conduct for contracting parties, including foreign investors (UAE Civil Transactions Law No. 25 of 2025, Art. 246).

In addition, the UAE legislator confirmed the principle of the binding force of contracts under the rule that “the contract is the law of the contracting parties”, meaning that a contract may not be cancelled or amended except by agreement between the parties or according to the law ( AlQodsi et al., 2024; Alenizi & Madi, 2019). This principle is considered one of the most important guarantees relied upon by the foreign investors, because it provides stability and clarity in contractual obligations, especially in contracts of an investment nature (UAE Civil Transactions Law No. 25 of 2025, Art. 113).

Furthermore, UAE’s civil law is based on the principle of freedom of contract, while taking into consideration the limits of public order and public morals. This principle gives the parties, including foreign investors, sufficient freedom to determine the contractual terms in a way that suits the nature of the investment activity, which provides flexibility in regulating investment relationships ( Al Tamimi, 2020; Abou Adel et al., 2025; Chami & Khater, 2025).

The civil transaction law also provides rules related to contractual and tort liability, which allow the parties to a contract and investors to claim compensation for damages resulting from breaching of contractual obligations were harmful acts. However, this protection is still based on general civil law rules and is not specifically designed to address the special nature of international investment disputes. This raises questions about whether these rules are sufficient to provide the required protection in some international investment disputes.

At the same time, the UAE legislator has worked on improving the attractiveness of the investment environment by allowing foreign investors to own up to 100% of companies and many economic sectors, after this ride had previously been restricted. This development has contributed to expanding investment opportunities within the state. In addition, the federal decree law No. 32 of 2021 regarding the Commercial Companies law aims to support the business environment and encourage the flow of foreign investment ( UAE Federal Decree-Law No. 32 of 2021; Muchlinski, 2021; Asif et al., 2025).

Based on this direction, modern legislation has granted foreign investors greater flexibility in establishing companies in different legal forms, while reducing the procedural restrictions that are related to incorporation and operation, and removing the requirement of having a national partner in many economic activities. The state has also adopted regulatory facilities in different economic sectors ( Zu, 2025), Including trade, industry, and services, while keeping some strategic activities subject to special controls. This reflects an approach that seeks to balance investment openness with the protection of national economic interests ( Van Harten, 2020).

These guarantees are consistent with several well-established standards in international investment law, most importantly that property cannot be expropriated except for public benefit and in return for fair compensation, in addition to the principle of fair and equitable treatment (FET) and the protection of legitimate expectations ( Dolzer & Schreuer, 2012).

The effectiveness of these guarantees has also been strengthened more by the accession of the UAE to several bilateral international agreements related to the protection and promotion of investment, as well as the adoption of procedural principles established by the United Nations Commission on International Trade Law (UNCITRAL).

Free zones have also played an important role in strengthening the investment environment by providing an independent regulatory system characterised by easy business establishment procedures, full foreign ownership, and tax exemptions and incentives granted to foreign investors within free zones for either limited or unlimited periods depending on the nature of the activity ( Muchlinski, 2021). This improves the efficiency of investment operations, reduces operational costs, and increases the competitiveness of the state as a global commercial centre.

Despite the significant development achieved by UAE legislation regulating foreign investment, the protection it provides remains general in some aspects because it was not specifically designed to regulate international investment relationships involving a foreign element. Therefore, the importance of benefiting from the rules of international investment law becomes clear to complete this protection and provide a higher level of legal guarantees for foreign investors while preserving the regulatory authority of the state.

### Legal protection of foreign investors under international investment law

International investment law represents the developed legal system for providing effective legal protection for foreign investors. It is based on a group of stablished legal principles and standards found in international agreements and investment arbitration decisions, which aim to protect foreign investments from arbitrary or unlawful actions that may be taken by the host state ( Peat et al., 2025; Dolzer & Schreuer, 2012).

This is linked to the principle of legitimate expectations protection as well, which is considered one of the key elements of the fair and equitable treatment standard. Under this principle, the host state is expected to refrain from adopting legislative or regulatory measures that undermine the existing legal positions unjustifiably, thereby supporting the stability of the international investment relationships.

The Investment arbitration awards, and the legal scholarships have consistently emphasized the importance of clear and stable legislation on which the investors rely on when making investment decisions. They also highlighted the need to avoid sudden changes that could significantly affect an investor’s legal or economic position ( Dolzer & Schreuer, 2012; Qutieshat et al., 2026; Ibrahim et al., 2026).

In addition to the substantive guarantees, Investment dispute settlement mechanisms represent one of the most important elements of international investment law protection. These mechanisms give the foreign investors the right to bring there are disputes before independent and impartial arbitration centers to resolve disputes that may arise with the host state ( Sachs, Johnson & Coleman, 2022).

One of the most significant arbitration bodies is the International Centre for Settlement of Investment Disputes (ICSID), that was established under the Washington Convention of 1965. The ICSID Was created to provide a specialized legal mechanism for investment disputes resolution between the foreign investors and the host states ( Schreuer, 2009; Marcoux et al., 2024).

Although the international investment arbitration is considered a very effective method for investment disputes resolution ( Marcoux et al., 2024), some legal scholars argue that excessive reliance on it may, in certain cases, limit a state’s ability to exercise It’s regulatory authority in protecting the public interest. For example, Environmental Protection on public health. This concern is commonly referred to as the concept of the states “regulatory space” ( OECD, 2023; Nggilu et al., 2025).

This issue has increasingly become important, due to the broad interpretation of certain standards related to international investment protection, particularly the principle of fair and equitable treatment. Such broad interpretations may grant arbitral tribunals Are significant discretionary power, which can lead to inconsistent outcomes in cases involving similar facts ( Dolzer & Schreuer, 2012).

Furthermore, the absence of binding precedent system in international investment arbitration may lead to different arbitral decisions in similar cases. This raises concerns regarding the legal certainty and the predictability of the outcomes for both the investors and the host state.

In addition, there is an ongoing academic debate on balancing between the foreign investors’ protection, and the preservation of a state’s right to exercise its regulatory authority. Some scholars argue that an expansive interpretation of certain protection standards can restrict the state’s freedom to regulate sectors with sensitive nature.

Moreover, investment arbitration proceedings are often associated with high costs and lower levels of transparency, compared to the national courts. This may create significant financial and procedural burdens, particularly in high value investment disputes ( Sornarajah, 2021; Van Harten, 2020).

### Integration between national protection and international standards for foreign investors protection

By analyzing the UAE’s national legislations, particularly the Civil Transaction Law, the Foreign Direct Investment Law, and the Commercial Companies Law, the analysis shows that the UAE legislator has taken clear steps toward creating an attractive legal environment for foreign investment. This has been achieved through the adoption of flexible legal principles governing contracts, the expansion of foreign ownership rights, and the simplification of procedures for establishing companies and carrying out business activities.

While the UAE’s Civil Transactions Law sets out the general rules that are governing the contractual obligations, such as the principles of good faith and the binding force of contracts, the/foreign Investment Law, and the Commercial Companies Law focus more specifically on regulating the entry of foreign investors and facilitating their business activities within the country. This reflects a degree of integration between the general legal framework, and the specialized rules that govern investment ( Muchlinski, 2021; Zu, 2025; Madi & Masn, 2024).

At the international level, the International Investment Law, and Investment Arbitration standards provide a range of legal protections for foreign investors, including fair and equitable treatment, and protection against unlawful expropriation. In addition, they allow investors to resort to international arbitration to resolve disputes that may arise between the investor and the host state. ( Peat et al. 2025; ICSID, 1965; UNCTAD, 2023).

By comparing the national and international legal frameworks, the comparison shows that the UAE’s legislation provides general and flexible protection for foreign investors. The Civil Transaction Law is based on fundamental contractual Principles that offer a broad level of protection. However, this protection remains general naturally and does not include detailed safeguards specifically those designed for international investment. In contrast, the Foreign Direct Investment Law provides more specialized incentives and guarantees for foreign investors. At the international level, international investment law offers broader and more detailed protection that further strengthen the legal position for the foreign investors.

The interaction between the national and the international rules contributes to the stability of the investment transactions and enhances the attractiveness of the UAE’s investment environment. In this context, investment arbitration plays an important role as a legal mechanism that connects the national laws with the international standards, by balancing the protection of foreign investors with the states sovereign right to regulate its economic activities ( Peat et al. 2025; Sornarajah, 2021).

Therefore, it can be argued that the relationship between the national and the international systems is based on a degree of integration. However, achieving a greater legislative harmony requires more clarity regarding certain concepts, particularly those related to foreign investment, to reduce reliance on judicial or arbitral interpretation when determining the scope of the legal protection ( Imad et al., 2026).

The importance of this matter becomes more apparent when an investment dispute arises. In such circumstances, the foreign investors may rely on the protectionist standards contained in the international investment agreement, while the host state may rely on the provisions of its domestic law. As a result, investment arbitration remains the most suitable mechanism for balancing the interests of investors with the state’s right to preserve its economic sovereignty ( Sornarajah, 2021).

### Practical challenges in protecting foreign investors between the national legal

Although UAE legislation provides various legal and procedural safeguards to protect foreign investors, In addition to allowing access to the international investment arbitration mechanisms for dispute resolution ( Sachs, Johnson & Coleman, 2022), Evaluating the effectiveness of this protection, can reveal some practical challenges arising from interaction between the national legal system, and the rules of international investment law.

One of the main challenges is the absence of a unified legislative definition of the foreign investor protection, which can affect the clarity of the scoop and limits of the legal protection. In addition, the legal provisions governing investor protection are scattered across several laws, rather than being consolidated then a single legislative framework, which can be easily referred to when it is needed. This creates practical difficulties in identifying a clear legal regime that fully protects the rights of foreign investors.

However, these observations do not undermine the effectiveness of the protection provided under the UAE legislation or the importance of international investment arbitration. Rather, they highlight the areas that could be further developed, to improve the clarity of the legal rules, and enhance legal certainty, in line with the modern trends in international investment law.

In this context, it is important to examine the relationship between judicial and arbitral practice in the UAE, and the challenges that may affect the International Investment Dispute Settlement System (ISDS). While at the national level, the UAE course generally support the principles of transactional stability, and the binding force of contracts by applying the principle of ““the contract is the law of the parties.” Courts usually avoid interfering with the parties contractual intentions, except in limited circumstances, particularly where issues of public policy, or defects and consent arise.

This approach reflects respect for the principles of legal certainty, and legitimate expectations in the commercial transactions, which are consistent with modern international standards for investment protection.

Booth the Civil Transactions Law, And the Commercial Transactions Law, provide a strong legal foundation that supports investment in the UAE. Where the former promotes stability in the legal transactions across the country for all the parties concerned, while the latter offers the speed and flexibility required for commercial activities. This aligns with the need for international investment and enhances the legal confidence of the foreign investors.

Similarly, the Foreign Direct Investment Law has expanded the range of economic activities that are open to foreign investment, and introduced a few guarantees related to the property rights’ protection, as well as the freedom to transfer profits, and the capital without restrictions. These measures provide foreign investors with a high level of financial security, and reduce the risks associated with limitations on liquidity or profits generated from investment activities, which is consistent with the requirements of the international investment ( Alhajaya, Madi, & Kameel, 2022).

Furthermore, the UAE Commercial Companies Law No. (32) of 2021 Brought significant developments to the investment environment, by allowing full foreign ownership in many economic sectors. This has given the foreign investors greater administrative and financial independence, while also simplifying the company incorporation and governance procedures.

Additionally, the competent regulatory authorities have played an important role in accelerating procedures and reducing administrative complexity. Examples include the Department of Economy and Tourism in Dubai, and the Abu Dhabi Department of Economic Development, whose efforts have contributed to improving the efficiency of the investment transactions across the UAE.

At the institutional level, the arbitration environment in the UAE is considered generally supportive of alternative dispute resolutions mechanisms. USA courts have adopted the principle of respecting arbitration agreements, and limiting judicial intervention in the arbitral awards, which enhances the effectiveness of arbitration, as a primary method for investment-related disputes resolution. In addition, the arbitration centers such as Dubai International Arbitration Centre (DIAC), And the Abu Dhabi arbitration institutions, play an important role and strengthen confidence in the national legal system, particularly in disputes that involve foreign parties.

However, despite this progress at the national level, there are several challenges remaining within the International Investment Dispute Settlement System (ISDS). Although the ISDS plays an important role in protecting the investors, concerns continue to exist regarding the inconsistent interpretation of certain substantive standards, especially the fair and equitable treatment standard. This standard remains open to different interpretations by the arbitral tribunals, which may lead to different legal outcomes in cases involving similar facts ( UNCTAD, 2023).

Therefore, it can be argued that the UAE’s legislative policy has successfully combined economic openness with strong legal safeguards, contributing to the stability of the investment environment, and the protection of the foreign capital. This approach has strengthened the UAE’s position as an attractive destination for foreign investment ( Van Harten, 2020).

It can be clearly seen that the relationship between the judicial, and the arbitral system in the UAE and the international investment arbitration system, is not one of separation, but rather one of mutual interaction. This connection reflects the ability to balance the protection of foreign investors, with the state’s right to regulate its economic affairs. Nevertheless, some of the practical challenges still require attention, particularly the need for greater procedural clarity in certain sections, to ensure a higher degree of consistency and legal protection.

At the same time, an ongoing challenge remains in maintaining a balance between continuing to attract foreign investment, and safeguarding national economic interests, especially in the light of the increasing regional and international competition. ( Van Harten, 2020).

### Comparative approaches and the development of the legal framework for foreign investor protection in the UAE

In the process of assessing the effectiveness of the UAE legal system and protecting foreign investors, it is valuable to consider the competitive experiences, particularly those of Singapore and France. Because these two countries provide different models for regulating foreign investment, while balancing the attraction of investment with the protection of the national interests.

On the one hand, the investment legal framework in Singapore is based on clarity, simplicity, and easy access to the rules governing economic activities. At the same time, it provides stable guarantees for the investors, including property rights protection, the freedom to transfer profits, and the legislative stability. Singapore has also been successful in reducing investment disputes through a clear regulatory environment, and by promoting alternative dispute resolution mechanisms, particularly mediation and commercial arbitration. This has increased the efficiency of the investment environment and increased legal certainty for investors. ( World Bank, 2020; Farrington et al., 2025).

On the other hand, France follows a different model that seeks balancing the investment openness with regulatory oversight. Certain foreign investments, particularly those involving strategic sectors, are subject to review and require prior approval procedures. This approach is intended to protect the state’s strategic interests and reflects A broader European trend toward safeguarding “economic sovereignty”, while continuing to comply with the international standards for investor protection. ( OECD, 2023).

While at the level of investment arbitration, both Singapore and France have worked to develop their investment dispute settlement systems ( Marcoux et al., 2024), with a particular focus on transparency, and the consistent interpretation of legal rules. Both countries have supported reforming efforts within the United Nations Commission on International Trade Law (UNCITRAL), Aimed at reducing inconsistencies in arbitral decisions and increasing confidence in the system.

When these models are compared with the UAE experience, it becomes clear that the UAE is closer to the Singaporean model in many respects, particularly in its adoption of an investment-friendly approach based on economic openness and regulatory flexibility. However, the UAE legal system could further benefit from improving legislative consistency and providing greater clarity in the legal definitions and concepts related to investor protection. At the same time, the UAE may draw lessons from the French experience by introducing more clearly defined safeguards for strategic and sensitive sectors without reducing the attractiveness of foreign investment.

This comparison demonstrates that enhancing the effectiveness of the UAE legal framework for the protection of foreign investors may be achieved by combining the clarity and flexibility of the Singaporean model with the balanced regulatory approach of the French model. Such an approach would contribute to a more stable investment environment and strengthen the UAE’s ability to keep pace with modern developments in international investment law.

Therefore, developing the legal framework for the protection of foreign investors in the United Arab Emirates does not require fundamental changes to the existing legislative system. Rather, it requires specific legislative and regulatory improvements aimed at increasing clarity and consistency, while also enhancing regulatory and procedural mechanisms in line with recent developments in international investment law ( Gharaibeh et al., 2024).

In this regard, there is a need to unify the legal definitions and concepts related to foreign investor protection, particularly the principles of fair and equitable treatment and the protection of legitimate expectations. This can be achieved either through amendments to existing legislation or by issuing an official interpretative guide that promotes transparency and reduces differences in the interpretation of these concepts.

On the other hand, recent developments in international investment law highlight the importance of consolidating and organizing the rules governing foreign investment within a unified legislative framework. Such an approach would make it easier for investors to identify and understand the legal rules applicable to foreign investment without having to navigate multiple laws, while preserving the flexibility that characterizes the UAE legal system.

In addition, increasing attention should be given to achieving a greater balance between investor protection and the state’s right to regulate. This can be accomplished by introducing clearer provisions in bilateral investment agreements that expressly recognize the state’s right to adopt regulatory measures necessary to protect the public interest, without such measures being considered a breach of its international obligations relating to investment protection.

With regard to the settlement of investment disputes, there is a need to strengthen transparency in investment arbitration proceedings by encouraging the publication of arbitral awards and applying the transparency rules developed by the United Nations Commission on International Trade Law (UNCITRAL). This would contribute to the development of more consistent legal principles and improve the predictability of arbitral decisions.

The development of legal protection for foreign investors also requires greater support for alternative dispute resolution methods, such as mediation and conciliation, before resorting to investment arbitration. This approach can help reduce the time and costs associated with international investment disputes.

In addition, it is important to enhance national institutional and judicial capacity in the field of investment disputes through specialized training for judges and arbitrators, as well as by strengthening the expertise of national legal professionals in handling investment disputes at both the domestic and international levels.

In light of the above, it is evident that adopting these proposals would improve the clarity of legal rules and achieve a greater balance between the protection of foreign investors and the state’s right to regulate its public policies. This, in turn, would support the stability of the investment environment in the United Arab Emirates and reinforce its position as an attractive destination for foreign investment.

## Conclusion

The study concluded that the legal system of the United Arab Emirates provides a significant level of legal protection for foreign investors and that this protection is reasonably effective in regulating investment relationships of a contractual nature. However, there remains room for further development in light of the rapid changes taking place in the international investment environment and the continuing evolution of investment protection standards. This is particularly important given the economic and regional developments witnessed in the Gulf region in recent period, which have highlighted the practical importance of having clear and stable legal rules when making long-term investment decisions.

The study also found that the relationship between national legislation and the rules of international investment law is not based on complete alignment between the two systems. Rather, it is based on a degree of practical integration that allows for broader legal protection of foreign investors while preserving the state’s regulatory authority. While national legislation provides the legal foundation for the stability of transactions and contractual relationships within the state, international investment law offers more specialized guarantees concerning investment protection standards and the settlement of investment disputes.

The study further demonstrated that the UAE legal system has achieved significant progress in regulating the legal framework for foreign investment and has become closely aligned with many modern standards of international investment law. However, practical application still reveals the need for greater clarity regarding certain legal concepts related to investment protection, as well as a reduction in differences in the interpretation of some legal rules. Addressing these issues would contribute to a higher level of legal certainty and greater stability in investment relationships.

In light of these findings, the study emphasizes the importance of continuing to develop certain legislative and regulatory mechanisms related to foreign investment. Such efforts would strengthen consistency between national legislation and international investment standards, enhance confidence in the UAE investment environment, and reduce the likelihood of investment disputes arising in the future.

### Recommendations

• Enhancing and strengthening the coordination and integration between the national legislation, and the international obligations that are related to the protection to foreign investment to reduce potential conflicts, and enhance the clarity of the legal framework
• Further developing the legal provisions that are governing foreign investment in manner that reflects the specific nature of the international investment relationships and keeps pace with the recent developments in international investment law.
• Promoting alternative methods for investment disputes resolution, particularly mediation and arbitration, While working on reducing their costs to ensure the timely and effective resolution of disputes.
• Providing clear legal guidance and informational materials for foreign investors to acknowledge them about their rights and obligations within the state, which would help to reduce disputes and promote contractual stability.
• Establishing clearer interpretative mechanisms for concepts related to foreign investor protection comma thereby achieving greater consistency between The national implementation and the international investment standards.

## Ethical considerations

Not applicable.

## Data Availability

This study is based on doctrinal legal analysis and publicly available sources. No new datasets were generated.

## References

[ref1] Adel M a A Elhalafawy AI Khodier GA : Unsettling the norm: a posthumanist reading of sameness and differences in Kathryn Erskine’s Mockingbird. *Theory and Practice in Language Studies.* 2025;15(11):3511–3520. 10.17507/tpls.1511.03

[ref2] Al Tamimi H : *UAE Civil Transactions Law and Contract Principles.* Dubai Legal Studies Publications;2020.

[ref3] Alenizi Z Madi R : The compatibility of Arab laws and modern international trends with the freedom of parties to choose the law applicable to international trade contracts. *Global Trade and Customs Journal.* 2019;14(9). 10.54648/GTCJ2019050

[ref4] Alhajaya NHA Madi R Kameel T : Analysis of the main problems and issues in arbitration of the foreign investment disputes: The concept of investment dispute and multiplicity of arbitration disputes relating to the same investment. *International Journal of Economics and Business Research.* 2022;27(1):93–110. 10.1504/IJEBR.2024.136155

[ref5] AlQodsi EM Eissa N Jadalhaq IM : Can UAE contract law accommodate legal capacity challenges posed by autism spectrum disorders?. *Social Sciences & Humanities Open.* 2026;13:102554. 10.1016/j.ssaho.2026.102554

[ref6] AlQodsi EM Jadalhaq IM El Maknouzi MEH : Suspension of the statute of limitations for civil claims: A comparative study of Arab legislations. *Social Sciences & Humanities Open.* 2024;10:101129. 10.1016/j.ssaho.2024.101129

[ref7] Asif M Lahiani H Alhourani MI : Honor killing and the cycle of social influence: A study of the Pakistani bride through Albert Bandura’s social learning theory. *Research Journal in Advanced Humanities.* 2025;6(2):1–14. 10.58256/pgj52m46

[ref8] Chami Y Khater MH : Re-evaluating contractual relativity: Third-party effects in Islamic and positive legal frameworks. *Mazahib Jurnal Pemikiran Hukum Islam.* 2025;24(2):309–328. 10.21093/mj.v24i2.11151

[ref9] Chami Y Imad D Khater MH : Abuse of rights: A comparative study between Mālikī jurisprudence & positive law. *AHKAM: Jurnal Ilmu Syariah.* 2025;25(2):415–428. 10.15408/ajis.v25i2.44606

[ref10] Dolzer R Schreuer C : *Principles of International Investment Law.* Oxford University Press; 2nd ed 2012.

[ref11] El-Erian M Imad D Sulaiman S : The Role of Artificial Intelligence in Enhancing Corporate Governance and Achieving Sustainable Development. *Access to Justice in Eastern Europe.* 2026;9(1):208–232. 10.33327/AJEE-18-9.1-a000177

[ref12] Farrington F Jevremović N : Between a rock and a hard place: The impact of replacing or abolishing ISDS on investment-affected parties. *Journal of International Dispute Settlement.* 2025;16(3). 10.1093/jnlids/idaf023

[ref13] Gharaibeh ZIY Alazzam FAF Salih AJ : Examining challenges and prospects associated with implementing legal protections for electronic governances: A generic perspective from developing country. *Brazilian Business Review.* 2024;21(5). 10.15728/bbr.2023.1773.en

[ref14] Ibrahim A Gourari FM AbdelAziz GM : Artificial intelligence in criminal justice governance: Opportunities and legal challenges (A comparative study). *Corporate Law & Governance Review.* 2026;8(2):77–88. 10.22495/clgrv8i2p7

[ref15] Imad D Khater M Abouhaiba N : Doctrinal and Comparative Assessment of GMO Liability Laws in the UAE. *Journal of Human Rights, Culture and Legal System.* 2026;6(1):31–58. 10.53955/jhcls.v6i1.701

[ref16] International Centre for Settlement of Investment Disputes (ICSID): *Convention on the Settlement of Investment Disputes between States and Nationals of Other States.* World Bank Group;1965.

[ref17] Khater M Chami Y Imad D : Assessing the impact of green tourism on sustainable development: A case study of the United Arab Emirates. *Research Journal in Advanced Humanities.* 2025a;6(2). 10.58256/0m0wvr07

[ref18] Khater M Chami Y Albakjaji M : Legal Environmental Protection and Sustainable Development in the United Arab Emirates. *Journal of Human Rights, Culture and Legal System.* 2025b;5(2):378–411. 10.53955/jhcls.v5i2.469

[ref19] Lashchykhina V Lashchykhina I : Protection of the rights and legitimate interests of foreign investors in Ukraine. *The Scientific Heritage.* 2024;148(148):19–23. 10.5281/zenodo.14063608

[ref20] Madi R Masn M : The effects of the public joint stock companies’ conversion on partners and creditors of the company: A comparative study between the Emirati and the Jordanian laws. *Vestnik of Saint Petersburg University Law.* 2024;15(1):235–249. 10.21638/spbu14.2024.115

[ref21] Marcoux J-M Bjorklund AK Whitsitt EA : Discourses of ISDS reform: A comparison of UNCITRAL Working Group III and ICSID processes. *J. Int. Econ. Law.* 2024;27(2):314–335. 10.1093/jiel/jgae017

[ref22] Muchlinski P : *Multinational Enterprises and the Law.* Oxford University Press; 3rd ed 2021. 10.1093/law/9780198824138.001.0001

[ref23] Nggilu NM Chami Y Khater M : Constructing Humanitarian-Based Law: A Philosophical Analysis of the Philanthropic Legal Paradigm. *Yustisia.* 2025;14(3):283–311. 10.20961/yustisia.v14i3.98924

[ref24] Organization for Economic Co-operation and Development (OECD): *OECD Investment Policy Reviews: United Arab Emirates.* OECD Publishing;2023.

[ref25] Peat D Hailes O Zhao C : Mismatched commitments: Treaty law solutions for multilateral ISDS reform. *J. Int. Econ. Law.* 2025;29(1):2–19. 10.1093/jiel/jgaf040

[ref26] Qutieshat E Al Adwan M Alshibli AA : Governing legal chatbots: Ethics, professional responsibility and liability in comparative perspective. *Research Journal in Advanced Humanities.* 2026;7(1). 10.58256/ppfy4k96

[ref27] Sachs L Johnson L Coleman J : *Yearbook on International Investment Law & Policy.* Oxford University Press;2022.

[ref28] Schreuer C : *The ICSID Convention: A Commentary.* Cambridge University Press; 2nd ed 2009. 10.1017/CBO9780511596896

[ref29] Sornarajah M : *The International Law on Foreign Investment.* Cambridge University Press; 5th ed 2021. 10.1017/9781316459959

[ref30] UAE Federal Decree-Law No. 32 of 2021 on Commercial Companies.

[ref31] UAE Federal Law No. 19 of 2018 on Foreign Direct Investment (as amended by Federal Law No. 16 of 2020).

[ref32] UAE Civil Transactions Law No. 25 of 2025 (Federal Decree-Law No. 25 of 2025). Official Gazette, No. 809 (Supplement 2).

[ref33] United Nations Conference on Trade and Development (UNCTAD): *World Investment Report 2023: International Tax Reform and Sustainable Investment.* United Nations;2023. 10.18356/9789210027847

[ref34] Van Harten G : *The Trouble with Foreign Investor Protection.* Oxford University Press;2020. 10.1093/oso/9780198866213.001.0001

[ref35] World Bank: *Doing Business 2020: Comparing Business Regulation in 190 Economies.* World Bank Group;2020.

[ref36] Zu W : Investment Facilitation and the Structural Evolution of International Investment Law. *World Trade Rev.* 2025;24(5):543–565. 10.1017/S1474745625101110

