# Supplementary material for: Does the Legal Framework of the United Arab Emirates Comply with International Standards for the Protection of Foreign Investors?
Source: F1000Res. 2026 Jul 4;15:1069. [Version 1] doi: 10.12688/f1000research.184809.1 (PMC13644843; doi:10.12688/f1000research.184809.1)
Supplement: Peer review history

# Reviewer report: Reviewer response for version 1

**DOI:** 10.5256/f1000research.203990.r501122

**Published:** 2026-08-05

**Authors:** Rodrigo Polanco

**Affiliations:** 1 University of Bern, Bern, Canton of Bern, Switzerland

The article accurately describes the recent changes to UAE domestic legislation aimed at providing legal protection for foreign investors and briefly compares this protection with that offered by international investment agreements and the domestic legislation of Singapore and France.

 However, I believe that the article lacks a more detailed analysis, and the authors fail to address several underlying questions revealed by their research.

**For example:**

- What were the reasons behind the changes in UAE laws? What was the timeframe of these changes? Were these changes inspired by other countries' legislation? Are there any challenges in the implementation of these new laws?
- What is the average content of the UAE's international investment agreements (IIAs)? Why has the UAE signed so many of these agreements in recent years, becoming the third country with the most IIAs signed? Have any of the provisions included in them (e.g. fair and equitable treatment) caused controversy domestically?
- What is the UAE's experience of investor-state arbitration cases? What lessons can be learned from the seven publicly known cases? Have these cases influenced changes in domestic laws or investment treaty-making?
- Why were France and Singapore selected as comparators for the UAE? Why not other, more similar countries (for example, GCC countries)? The authors should provide a more detailed explanation of the reasons behind their selection. If they wish to pursue this comparison, it should delve deeper into the elements being compared; currently, it is rather superficial. 

Addressing these questions could make the article more analytical and less descriptive. Additionally, the text would benefit from proofreading and streamlining to avoid repetition.

Is the work clearly and accurately presented and does it cite the current literature?

Partly

If applicable, is the statistical analysis and its interpretation appropriate?

Not applicable

Are all the source data underlying the results available to ensure full reproducibility?

No source data required

Is the study design appropriate and is the work technically sound?

Partly

Are the conclusions drawn adequately supported by the results?

Partly

Are sufficient details of methods and analysis provided to allow replication by others?

Partly

Reviewer Expertise:

international investment law

I confirm that I have read this submission and believe that I have an appropriate level of expertise to confirm that it is of an acceptable scientific standard, however I have significant reservations, as outlined above.

# Reviewer report: Reviewer response for version 1

**DOI:** 10.5256/f1000research.203990.r501119

**Published:** 2026-07-20

**Authors:** Dr. Ramzi Madi

**Affiliations:** 1 Al Ain University, Al Ain, Abu Dhabi, United Arab Emirates

The manuscript examines a significant and timely issue by evaluating the extent to which the legal framework of the United Arab Emirates aligns with international standards governing the protection of foreign investors, particularly in the context of recent legislative reforms and the increasing importance of international investment arbitration. The study offers clear academic merit through its comparative analytical approach, its thorough assessment of UAE legislation in relation to international investment law, and its critical analysis of the relationship between domestic legal provisions and internationally recognized standards of investor protection. The manuscript is well organized, presents coherent and logically developed arguments, and draws conclusions that are firmly supported by the analysis. In addition, the study makes a valuable contribution to the existing body of knowledge while providing practical insights and recommendations that are relevant to both academics and policymakers. Overall, the manuscript satisfies the scientific and methodological criteria expected for indexing, and no substantive revisions are required. Accordingly, I recommend that it can be accepted for indexing in its present form without revision.

Is the work clearly and accurately presented and does it cite the current literature?

Yes

If applicable, is the statistical analysis and its interpretation appropriate?

Not applicable

Are all the source data underlying the results available to ensure full reproducibility?

Yes

Is the study design appropriate and is the work technically sound?

Yes

Are the conclusions drawn adequately supported by the results?

Yes

Are sufficient details of methods and analysis provided to allow replication by others?

Yes

Reviewer Expertise:

Commercial LawIntellectual Property LawInternet and Cyber LawArbitration Law Artificial Intelligence and LawE-commerce LawBiotechnology Law

I confirm that I have read this submission and believe that I have an appropriate level of expertise to confirm that it is of an acceptable scientific standard.

# Reviewer report: Reviewer response for version 1

**DOI:** 10.5256/f1000research.203990.r501123

**Published:** 2026-07-16

**Authors:** Nayel AlOmran

**Affiliations:** 1 Zayed University - Abu Dhabi Campus, Abu Dhabi, United Arab Emirates

**Manuscript Title:** *Does the Legal Framework of the United Arab Emirates Comply with International Standards for the Protection of Foreign Investors?*

 The manuscript addresses a highly significant and timely topic, particularly in light of the ongoing global and regional economic transformations, the increasing competition among states to attract foreign direct investment (FDI), and the growing reliance on international investment arbitration as a principal mechanism for resolving investment disputes. Considering the United Arab Emirates' continuous legislative reforms and its strategic objective of fostering a stable, transparent, and investor-friendly legal environment, the subject of this study is both relevant and of considerable academic and practical importance.

 The manuscript is well organized, clearly written, and demonstrates a strong command of the legal issues under examination. The comparative analytical approach is appropriate and effectively evaluates the extent to which the UAE legal framework aligns with internationally recognized standards for the protection of foreign investors. The analysis is rigorous, logically developed, and supported by relevant legal authorities and scholarly literature. The conclusions are well founded and follow coherently from the discussion presented.

 The study also makes a valuable contribution to the existing literature by offering a comprehensive assessment of the UAE's investment regime within the broader context of international investment law. Its findings will be of interest to academics, legal practitioners, policymakers, and researchers concerned with foreign investment protection and regulatory reform.

 As an observation for future research rather than a recommendation for revision, the authors may wish to further explore the practical implications of evolving geopolitical and economic developments in the Gulf region and their potential influence on the continued evolution of investment protection standards. Such an extension could provide an additional dimension to this important area of scholarship.

 Overall, this is a well-executed and Indexable manuscript that meets the standards of the journal.

**Recommendation:** **Accept without Revisions.**

Is the work clearly and accurately presented and does it cite the current literature?

Yes

If applicable, is the statistical analysis and its interpretation appropriate?

Not applicable

Are all the source data underlying the results available to ensure full reproducibility?

Yes

Is the study design appropriate and is the work technically sound?

Yes

Are the conclusions drawn adequately supported by the results?

Yes

Are sufficient details of methods and analysis provided to allow replication by others?

Yes

Reviewer Expertise:

Research Areas: Public International Law; Constitutional Law; Comparative Constitutional Law; Administrative Law; International Investment Law; Legal Studies.

I confirm that I have read this submission and believe that I have an appropriate level of expertise to confirm that it is of an acceptable scientific standard.
